# Assessing Patients’ Critical Health Literacy and Identifying Associated Factors: Cross-sectional Study

**DOI:** 10.2196/43342

**Published:** 2023-04-05

**Authors:** Yi Shan, Meng Ji, Zhaogang Dong, Zhaoquan Xing, Xiaofei Xu

**Affiliations:** 1 School of Foreign Studies Nantong University Nantong China; 2 School of Languages and Cultures, The University of Sydney Sydney Australia; 3 Department of Clinical Laboratory, Qilu Hospital of Shandong University Ji'nan China; 4 Department of Urology, Qilu Hospital of Shandong University Ji'nan China; 5 Center for Reproductive Medicine Department of Obstetrics and Gynecology Qilu Hospital of Shandong University Ji'nan China

**Keywords:** critical health literacy, Chinese patients, latent class modeling, limited critical health literacy, associated factors

## Abstract

**Background:**

Previous studies have revealed that functional health literacy plays a less important role than communicative and critical health literacy (CRHL) and that communicative literacy and CRHL contribute more to better patient self-management. Although improving health literacy has been identified as an approach to fostering community involvement and empowerment, CRHL may be regarded as the neglected domain of health literacy, rarely achieving any focus or interventions that claim to be working toward this outcome. Considering this research background, close scholarly attention needs to be paid to CRHL and its associated factors.

**Objective:**

This study aimed to assess CRHL and identify essential factors closely associated with the status of CRHL among Chinese patients and to provide some implications for clinical practice, health education, medical research, and public health policy making.

**Methods:**

We conducted this cross-sectional study, which lasted from April 8, 2022, to September 23, 2022, following the steps below. We first designed a 4-section survey questionnaire and then recruited Mandarin Chinese–speaking patients from Qilu Hospital of Shandong University, China, using randomized sampling. Subsequently, we administered the questionnaire via *wenjuanxing*, the most popular web-based survey platform in China, between July 20, 2022, and August 19, 2022. Finally, we used latent class modeling to analyze the valid data collected to classify the patient participants and identify the factors potentially associated with different CRHL levels.

**Results:**

All data in the 588 returned questionnaires were valid. On the basis of the collected data, we classified the patient participants into 3 latent classes of limited, moderate, and adequate CRHL and identified 4 factors associated with limited CRHL, including middle and old age, male sex, lower educational attainment, and low internal drive to maintain one’s health.

**Conclusions:**

Using latent class modeling, we identified 3 classes of CRHL and 4 factors associated with limited CRHL among the Chinese study participants. These literacy classes and the predicting factors ascertained in this study can provide some implications for clinical practice, health education, medical research, and health policy making.

## Introduction

### Background

Literacy in health information is becoming an essential factor that is essential for health status [1,2]. Health literacy is defined as “the capacity of an individual to obtain, interpret, and understand basic health information and services in ways that are health-enhancing” [3]. It is a major concern for health professionals and public health authorities [4]. Most studies used clinical cohorts that typically overrepresented socially disadvantaged groups, making it difficult to draw inferences regarding the overall status of health literacy in the general public [5]. It has been found that more than one-fourth of the 31,129 participants in 85 studies had *inadequate* health literacy, and another one-fifth had *marginal* health literacy [6]. Patients with low health literacy are likely to lack the skills essential for effectively interacting with the health system and engaging in appropriate self-care, including the practical knowledge about how to take medications and interpret labels and other health information [7]. Previous studies have linked limited health literacy to poorer health status, increased hospitalization, nonadherence to medications, medication dosing errors, and increased mortality [6,8-10].

Involving varying levels of cognitive, interpersonal, and social skills, the model of health literacy by Nutbeam [11,12] consists of 3 subsets of literacy: functional, communicative, and critical health literacy. Functional health literacy (FUHL) refers to the sufficient basic skills in reading and writing needed to function effectively in everyday situations, measuring a patient’s ability to perform basic reading and numerical tasks essential for functioning in the health care context [11]. Communicative health literacy (COHL) is defined as more advanced skills to actively participate in everyday activities, extract information, derive meaning from different forms of communication, and apply new information to changing situations [11]. Critical health literacy (CRHL) refer to higher-level cognitive and social skills that are essential for critically analyzing information and using this information to take individual and collective action for greater control over life events and situations and to appropriately handle social, economic, and environmental determinants of health [11]. CRHL can be divided into 3 components: critical analysis of information, understanding of the social determinants of health, and engagement in collective action [11,12]. To develop higher CRHL skills, individuals need to integrate knowledge regarding human health rights with health advocacy goals to take appropriate individual and collective health-related actions [13]. CRHL can also include self-efficacy for making decisions and supporting others in making appropriate health choices [14]. CRHL is potentially a higher-order process that can be developed through education to critically evaluate information relevant to health [15], and it is a process in which individuals become aware of issues, engage in critical dialogue, and become involved in health-related decision-making [16].

In the context of a growing body of research on health literacy, the World Health Organization as well as researchers and theorists involved in health promotion and public health have enthusiastically responded to this growth [17-19]. Researchers tend to approach health literacy either from the perspective of medicine to examine risk factors for health outcomes or from the perspective of health promotion and health education to deliver interventions to improve the clarity of health information [20]. Previous studies have revealed that FUHL plays a less important role than COHL and CRHL and that COHL and CRHL contribute more to better patient self-management [21-23]. Although improving health literacy has been identified as an approach to fostering community involvement and empowerment, CRHL may be regarded as “the neglected domain of health literacy, rarely achieving any focus or interventions that claim to be working towards this outcome” [23]. Considering this research background, close scholarly attention needs to be paid to CRHL and its associated factors.

### Objective

This study aimed to assess CRHL and identify significant factors closely associated with the status of CRHL among Chinese patients and to provide some implications for clinical practice, health education, medical research, and public health policy making.

## Methods

### Overview

We conducted this cross-sectional study that lasted from April 8, 2022, to September 23, 2022, following the steps given in subsequent sections. We first designed a 4-section survey questionnaire and then recruited Mandarin Chinese–speaking patients from Qilu Hospital of Shandong University, China, using randomized sampling. Subsequently, we administered the questionnaire between July 20, 2022, and August 19, 2022. Finally, we used latent class modeling to analyze valid data collected to classify the patient participants and identify factors potentially associated with different CRHL levels.

### Questionnaire Design

To reveal potential factors contributing to CRHL status, we incorporated the following information into the survey questionnaire: (1) age, sex, and education; (2) self-reported disease knowledge; (3) 3 validated health literacy assessment instruments (ie, All Aspects of Health Literacy Scale [AAHLS] [24], the eHealth Literacy Scale [eHEALS] [25], and the General Health Numeracy Test [GHNT]–6 [26]); and (4) the Multidimensional Health Locus of Control (MHLC) scales Form A [27]. The 12-item AAHLS consists of 3 subscales, including the COHL subscale, FUHL subscale, and CRHL subscale, which have been defined in the *Introduction* section. The 8-item eHEALS evaluates the study participants’ knowledge and skills that are essential for using eHealth resources and interventions. The 6-item GHNT-6 assesses patients’ understanding and capacity to act on numerical health information to help providers and educators tailor education to patients. Both the eHEALS and the GHNT-6 do not have subscales. The 18-item MHLC Form A comprises three 6-item subscales that measure “Internal,” “Chance,” and “Powerful Others” locus of control, that is, “beliefs that the source of reinforcements for health-related behaviors is primarily internal, a matter of chance, or under the control of powerful others” [28]. Such beliefs can motivate health behavior, which refers to taking voluntary actions to promote health, reduce health risks [29], and mediate health status [30,31]. Individuals categorized as having an “Internal” locus of control are more likely to engage in health behaviors and are more knowledgeable regarding their health problems [32,33]. Informed by relevant studies [7-16,28-33], we hypothesized that the participants’ CRHL status could be closely associated with their health literacy status measured by the AAHLS, eHEALS, and GHNT-6, and their health beliefs evaluated by the MHLC Form A.

### Participant Recruitment

The study participants were recruited from Qilu Hospital of Shandong University, China, using randomized sampling. Participants who had met the four inclusion criteria were invited to participate in this survey, and we included those: (1) being aged ≥18 years, (2) having at least primary education (Year 6 schooling) to understand the questions in the questionnaire, (3) being patients rather than relatives accompanying patients, and (4) participating in the survey voluntarily. We made face-to-face contact with Mandarin Chinese–speaking patients who were attending the outpatient clinic and those who were hospitalized to identify those who satisfied the inclusion criteria, explain them about the purpose of the survey, and ask them to participate in the web-based survey as scheduled. We identified 858 eligible patients.

### Questionnaire Survey and Data Collection

The survey lasted 1 month from July 20, 2022, to August 19, 2022. The questionnaire (Multimedia Appendix 1) was administered via *wenjuanxing* [34], the most popular web-based questionnaire platform in China. Participants filled out the administered questionnaire on the web. Returned questionnaires were considered valid only when all question items included were answered according to our predefined validation criterion.

On August 20, 2022, the returned questionnaires were downloaded in the format of an Excel file (Microsoft Corp) from *wenjuanxing*. A total of 588 answered questionnaires were returned, with a response rate of 68.5% (588/858). We double-checked the returned questionnaires and found all of them to be valid.

### Data Coding and Latent Class Analysis

To code valid data, we used predefined coding schemes based on Likert scales with varying score ranges for the different questionnaire items. We then used latent class analysis (LCA; Latent GOLD 5.0) to classify the patient participants into different clusters according to their CRHL status and identified factors potentially associated with their different CRHL levels.

LCA is increasingly applied in social and health sciences. LCA has methodological advantages over traditional clustering techniques [35-38]. A notable benefit of LCA is the probabilistic attribution of latent class membership to study participants using maximum likelihood estimation [35]. As a result, each observed participant attains a probability of belonging to a certain latent class. For example, within a 2-class LCA solution, a study participant can have 2 probabilities associated with either latent class. Within a 3-class LCA model, the participant can automatically have 3 probabilities indicating their likelihood of belonging to each of the 3 latent classes. The combined probabilities of class memberships sum to 1, based on the conditional independence assumption of LCA. The probabilistic nature of LCA adds to the complexity of the result interpretation. However, in practice, the more flexible, intuitive approach of LCA when compared with “hard, rigid” clustering techniques allows researchers more insights into the impact of predictor variables on latent class membership fluidity and dynamics, as well as the susceptibility of class memberships to the definition and selection of probability thresholds to suit different research purposes.

### Ethics Approval

This study was approved by the Ethics Review Board of Qilu Hospital of Shandong University, China. The review number is KYLL-202208-026. The study data were anonymized to protect the privacy and confidentiality of the study participants. Because the participants voluntarily participated in the survey to support and promote academic research, no compensation was provided for them as per the common practice in China.

## Results

### Descriptive Statistics

Table 1 presents the descriptive statistics of the data collected from the patient participants. All the data in the 588 returned questionnaires were valid. The patients had a mean age of 39.20 (SD 11.59) years. Of the 588 participants, 366 (62.2%) were female individuals. The mean score for education was 3.68 (SD 1.45), indicating that their average educational level was between Year 12 schooling and junior college. They assessed the status of their disease knowledge as between “knowing a lot” and “knowing some,” with a mean score of 2.53 (SD 0.90). The mean scores of the functional and communicative items in the AAHLS were as follows: 2.09 (SD 0.71), 3.04 (SD 0.90), and 2.15 (SD 0.73) for the 3 FUHL items and 1.61 (SD 0.72), 1.80 (SD 0.72), and 1.79 (SD 0.73) for the 3 COHL items. These mean scores indicate that they “sometimes” needed help to read and comprehend health information and to complete official documents but were “rarely” able to identify and secure others’ help. The average score of the 8 items on the eHEALS was approximately 3, with an SD of approximately 1.10, indicating uncertainty regarding their skills to use eHealth resources and interventions. The mean score for each item on the GHNT scale was 1.58 (SD 0.49), 1.25 (SD 0.43), 1.29 (SD 0.46), 1.89 (SD 0.31), 1.77 (SD 0.42), and 1.68 (SD 0.47), showing that a large proportion of participants answered the 6 questions on the GHNT scale incorrectly, especially questions 1 (348/588, 59.2%), 4 (525/588, 89.3%), 5 (453/588, 77%), and 6 (402/588, 68.4%). Regarding their scoring performance on the MHLC scales Form A, the participants scored averages of 21.15 (SD 5.65), 16.23 (SD 4.48), and 19.74 (SD 4.50) on the “Internal,” “Chance,” and “Powerful Others” subscales, respectively. The average score determined between responses of “slightly disagree” and “slightly agree” for the “Internal” subscale indicates that they were not sure of their internal drivers to maintain health. The average score determined between responses of “moderately disagree” and “slightly disagree” for the “Chance” subscale implies that they were generally less likely to attribute their health to a matter of luck. The average score determined between responses of “moderately disagree” and “slightly disagree” for the “Powerful Others” subscale means that they were generally uncertain about the role of others in the maintenance of their health.

**Table 1:** Descriptive statistics of the data collected (N=588).

Predictor variables			Values, mean (SD; range)
Age (years)			39.2 (11.59; 17-68)
Sex			N/A^a^
Education (years)			3.68 (1.45; 1-6)
Disease knowledge			2.53 (0.9; 1-4)
**FUHL^b^**			
	Item 1: How often do you need someone to help you when you are given information to read by your physician, nurse, or pharmacist?	2.09 (0.71; 1-3)	
	Item 2: When you need help, can you easily get someone to assist you?	3.04 (0.9; 1-4)	
	Item 3: Do you need help to fill in official documents?	2.15 (0.73; 1-3)	
**COHL^c^**			
	Item 1: When you talk to a physician or nurse, do you give them all the information they need to help you?	1.61 (0.72; 1-3)	
	Item 2: When you talk to a physician or nurse, do you ask the questions you need to ask?	1.8 (0.72; 1-3)	
	Item 3: When you talk to a physician or nurse, do you ensure they explain anything that you do not understand?	1.79 (0.73; 1-3)	
**eHL^d^**			
	Item 1: I know what health resources are available on the Internet.	2.92 (1.1; 1-5)	
	Item 2: I know where to find helpful health resources on the Internet.	3.03 (1.11; 1-5)	
	Item 3: I know how to find helpful health resources on the Internet.	3.05 (1.11; 1-5)	
	Item 4: I know how to use the Internet to answer my health questions.	3.1 (1.08; 1-5)	
	Item 5: I know how to use the health information I find on the Internet to help me.	3.06 (1.15; 1-5)	
	Item 6: I have the skills I need to evaluate the health resources I find on the Internet.	3.02 (1.13; 1-5)	
	Item 7: I can tell high quality from low-quality health resources on the Internet.	3.08 (1.1; 1-5)	
	Item 8: I feel confident using information from the Internet to make health decisions.	2.98 (1.1; 1-5)	
**GHNT^e^**			
	Item 1: Call your physician if you have a temperature of 100.4 °F or greater. The thermometer looks like the following: 100.2 F : Do you call a physician?	1.58 (0.49; 1-2)	
	Item 2: If 4 people out of 20 have a chance of getting a cold, what would be the risk of getting a cold?	1.25 (0.43; 1-2)	
	Item 3: Suppose that the maximum heart rate for a 60 year old woman is 160 beats per minute and that she is told to exercise at 80% of her maximum heart rate. What is 80% of that woman’s maximum heart rate?	1.29 (0.46; 1-2)	
	Item 4: You ate half the container of carrots. How many grams of carbohydrate did you eat?	1.89 (0.31; 1-2)	
	Item 5: Your doctor tells you that you have high cholesterol. He informs you that you have a 10% risk of having a heart attack in the next 5 years. If you start on a cholesterol-lowering drug, you can reduce your risk by 30%. What is your 5-year risk if you take the drug?	1.77 (0.42; 1-2)	
	Item 6: A mammogram is used to screen women for breast cancer. False positives are tests that incorrectly show a positive result. 85% of positive mammograms are actually false positives. If 1000 women receive mammograms, and 200 are told there is an abnormal finding, how many women are likely to actually have breast cancer?	1.68 (0.47; 1-2)	
Internal^f^ sum scores			21.15 (5.65; 6-36)
Chance^g^ sum scores			16.23 (4.84; 6-36)
Powerful Others^h^ sum scores			19.74 (4.5; 6-36)

^a^N/A: not applicable.

^b^FUHL: functional health literacy.

^c^COHL: communicative health literacy.

^d^eHL: Electronic Health Literacy.

^e^GHNT: General Health Numeracy Test.

^f^The Internal Locus of Control: beliefs that one’s health is up to their own actions and behaviors.

^g^The Chance Locus of Control: beliefs that one’s health is up to fate, chance, or luck.

^h^The Powerful Others Locus of Control: beliefs that one’s health is up to others’ actions and behaviors.

### Latent Class Modeling

#### Determination of 3 Latent Clusters

Tables 2 and 3 show the model fit statistics of the LCA. The Akaike information criterion (AIC) and Bayesian information criterion (BIC) provide measures of model performance, which are often used as key criteria to select the best-performing models. Both AIC and BIC are calculated for candidate models, and the “best” model is the candidate model with the smallest AIC and BIC. We also examined the Lo-Mendell-Rubin likelihood ratio test, also known as the Vong-Lo-Mendell-Rubin test, and the bootstrap likelihood ratio test [39,40] to determine whether models with more clusters (k+1) had statistically improved performance over the earlier k model. Small *P* values are indicators of better fitness of the (k+1) cluster model over the k-cluster model. Normalized entropy is another important measure of the model fitness. It measures the aggregated amount of classification uncertainty and ranges from 0 to 1. Entropies closer to 1 indicate better posterior classification performance of the probabilistic modeling, with a threshold of 0.8 as indicative of good model discrimination [41].

*BIC_L_^2^ = L^2^* − log*(N) df,*

*AIC_L_^2^ = L^2^* − 2 *df,*

*AIC3_L_^2^ = L^2^* − 3 *df,*

*CAIC_L_^2^ = L^2^* − (log*(N)* + 1) *df,*

*SABIC_L_^2^ = L^2^* − log ((*N* + 2) / 24) *df.*

These information criteria weight the fit and the parsimony of a model: the lower BIC, AIC, AIC3, CAIC, or SABIC the better the model.

**Figure 1:**
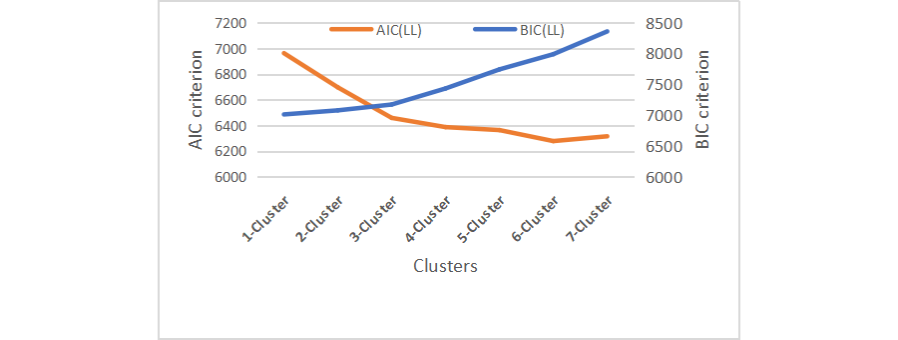
Changes in model fit statistics. AIC: Akaike information criterion; BIC: Bayesian information criterion; LL: log-likelihood.

We used the “elbow graph” method to determine the optimal number of clusters for the latent class modeling. Figure 1 shows that as the number of latent classes increased, the AIC decreased and BIC increased. The first sharp decrease in the AIC occurred with the 3-cluster solution. In addition, after the 3-cluster model, the BIC value increased more rapidly despite the AIC value continuing to decrease. More latent clusters would also increase the complexity of interpreting the model. We, therefore, set the optimal number of clusters at 3. As a result, we identified 3 latent classes of CRHL among the study participants.

Tables 4 and 5 show descriptive statistics of the 3 latent clusters representing the 3 levels of CRHL among the study participants. The Games-Howell test in Table 6 suggests that there were statistically significant differences among the 3 clusters.

**Table 2:** Model fit statistics (1).

Models	LL^a^	BIC^b^ (LL)	AIC^c^ (LL)	AIC3 (LL)	Npar^d^	L²^e^	*df* ^e^	*P* value^f^
1-cluster	−3470.26	7010.65	6962.51	6973.51	11.00	6940.51	577.00	<.001
2-cluster	−3261.06	7076.90	6696.12	6783.12	87.00	6522.12	501.00	<.001
3-cluster	−3066.08	7171.57	6458.16	6621.16	163.00	6132.16	425.00	<.001
4-cluster	−2954.16	7432.35	6386.31	6625.31	239.00	5908.31	349.00	<.001
5-cluster	−2866.60	7741.86	6363.19	6678.19	315.00	5733.19	273.00	<.001
6-cluster	−2747.72	7988.73	6277.43	6668.43	391.00	5495.43	197.00	<.001
7-cluster	−2690.39	8358.71	6314.78	6781.78	467.00	5380.78	121.00	<.001

^a^LL: log-likelihood (the smaller the absolute value of log-likelihood, the better the model fit).

^b^BIC: Bayesian information criterion (values closer to 0 indicate better fit).

^c^AIC: Akaike information criterion (values closer to 0 indicate better fit).

^d^Npar: number of estimated parameters.

^e^L^2^, *df*: the sample size*–*adjusted BIC (SABIC) based on the L^2^ and *df*, which is the more common formulation in the analysis of frequency tables. They are defined as:

^f^All *P* values <.01.

**Table 3:** Model fit statistics (2).

Models	Bootstrap p^a^	VLMR^b^	*P* value	−2 LL diff^c^	Bootstrap *P* value	Class error	Entropy *R*²^d^
1-cluster	0.36	N/A^e^	N/A	N/A	N/A	0.00	1.00
2-cluster	0.04	418.39	<.001	418.39	<.001	0.00	0.98
3-cluster	0.02	389.96	<.001	389.96	<.001	0.01	0.97
4-cluster	0.00	223.85	<.001	223.85	<.001	0.00	0.99
5-cluster	0.00	175.12	<.001	175.12	<.001	0.00	0.99
6-cluster	0.00	237.76	<.001	237.76	<.001	0.00	0.99
7-cluster	0.00	114.65	<.001	114.65	<.001	0.00	1.00

^a^Bootstrap *P* value: If *P*<.05, the k-class model is selected over the k-1 class model. Rather than relying on the asymptotic *P* value, it also possible to estimate the *P* value associated with the goodness-of fit chi-squared statistics by means of a parametric bootstrap.

^b^VLMR: Vuong-Lo Mendell-Rubin test. Use to test if a model with k classes is better than model with k-1 class (eg, a 3-class vs a 2-class model). We recommend conducting and reporting VLMR tests where applicable.

^c^−2 LL diff: −2 log-likelihood difference.

^d^Entropy *R*²: Values >0.8 indicate high degree of separation between classes.

^e^N/A: not applicable.

**Table 4:** Conditional probabilities of responses to items of critical health literacy (CRHL) of all aspects of Health Literacy Scale.

				Cluster 1		Cluster 2		Cluster 3
Overall				0.3770		0.3348		0.2881
**Indicators**								
	**CRHL1^a^**							
		1	0.7773		0.1574		0.0653	
		2	0.2513		0.2393		0.5095	
		3	0.0975		0.7302		0.1723	
	**CRHL2^b^**							
		1	0.7412		0.2153		0.0435	
		2	0.2428		0.2521		0.5050	
		3	0.1393		0.7595		0.1012	
	**CRHL3^c^**							
		1	0.6468		0.2353		0.1179	
		2	0.2720		0.2351		0.4929	
		3	0.1710		0.7584		0.0707	
	**CRHL4^d^**							
		1	0.6123		0.3366		0.0511	
		2	0.3301		0.2211		0.4488	
		3	0.2545		0.5512		0.1943	
	**CRHL5^e^**							
		1	0.6890		0.2599		0.0510	
		2	0.2945		0.2408		0.4647	
		3	0.2014		0.6039		0.1946	
	**CRHL6^f^**							
		Better information	0.4819		0.2143		0.3038	
		Better facilitates	0.2693		0.4587		0.2720	

^a^CRHL Item 1: Are you someone who likes to find out lots of different information about your health?

^b^CRHL Item 2: How often do you think carefully about whether health information makes sense in your particular situation?

^c^CRHL Item 3: How often do you try to work out whether information about your health can be trusted?

^d^CRHL Item 4: Are you the sort of person who might question your doctor or nurse’s advice based on your own research?

^e^CRHL Item 5: Do you think that there plenty of ways to have a say in what the government does about health?

^f^CRHL Item 6: What do you think matters most for everyone’s health? a) information and encouragement to lead healthy lifestyles; b) good housing, education, decent jobs and good local facilities.

**Table 5:** Descriptive statistics of the latent clusters.

Clusters	Participants (n=588), n (%)	Values, mean (SD; SE)
1	221 (37.6)	9.19 (1.33; 0.09)
2	197 (33.5)	12.91 (1.41; 0.10)
3	170 (28.9)	11.72 (0.91; 0.07)
Total	N/A^a^	11.17 (2.04: 0.08)

^a^N/A: not applicable.

**Table 6:** Multiple comparisons of intercluster differences.

Clusters (I) and (J)			Mean difference (I-J; SE; 95% CI)		*P* value
**1**					
	2	−3.71^a^ (0.13; −4.0281 to −3.3993)		<.001	
	3	−2.52353^a^ (0.11; −2.7887 to −2.2584)		<.001	
**2**					
	1	3.71371^a^ (0.13; 3.3993 to 4.0281)		<.001	
	3	1.19018^a^ (0.12; 0.9027 to 1.4776)		<.001	
**3**					
	1	2.52353^a^ (0.11; 2.2584 to 2.7887)		<.001	
	2	−1.19018^a^ (0.12; −1.4776 to −0.9027)		<.001	

^a^The mean difference is significant at the .05 level.

#### Profile of the 3 Latent Clusters

Table 4 shows the distribution of conditional probabilities of different responses (often, sometimes, and rarely) to each of the 6 items of the CRHL scale within each latent cluster. Because of the conditional independence assumption, the conditional probabilities of the 3-level responses within each latent cluster sum to 1. Responses of higher conditional probabilities within each of the 3 clusters help us to understand the profile of each cluster, as well as the main differences among the clusters. As shown in Table 4, study participants in cluster 2 were consistently more likely to choose the third response (“rarely”) across all the 6 items of the CRHL scale. They were also inclined to believe that “good housing, education, decent jobs, and good local facilities” matter most for everyone’s health, instead of “information and encouragement to lead healthy lifestyles” (CRHL, item 6). In contrast, study participants in cluster 1 were consistently more likely to choose the first response (“often”) to all the 6 items of the CRHL scale, suggesting a much higher level of CRHL overall. Study participants in cluster 2 preferred to choose the second response (“sometimes”) across CRHL questions and were likely believers of the importance of good “health information and encouragement to lead healthy lifestyles.” On the basis of the observed response patterns across study participants, we thus labeled the 3 clusters as adequate CRHL (cluster 1), moderate CRHL (cluster 3), and low critical literacy (cluster 2).

**Figure 2:**
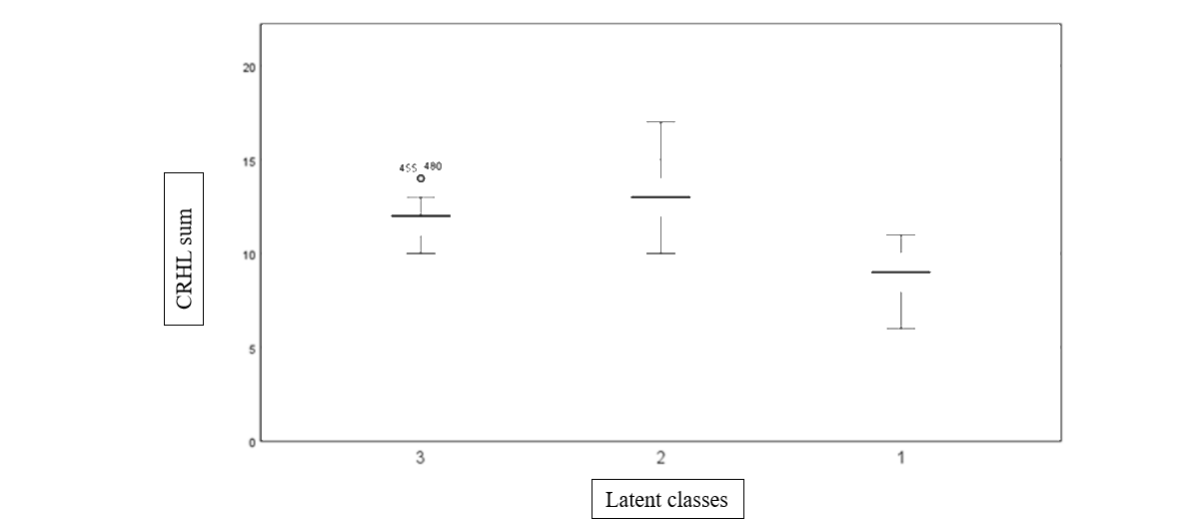
Boxplots of the sum of critical health literacy (CRHL) scores of the 3 latent classes.

As evident from Table 5 and Figure 2, class 2 had the highest average sum of CRHL (mean 12.91, SD 1.41). The content of the 6 items of the CRHL subscale of AAHLS is presented in the footnotes of Table 4. We coded the responses to these 6 items in the following manner: for the first, second, third, fourth, and fifth questions, we coded 1=often, 2=sometimes, and 3=rarely; for the last question, CRHL6, we coded 1=information and encouragement to lead healthy lifestyles and 2=good housing, education, decent jobs, and good local facilities. A higher sum of the response scores was thus indicative of lower CRHL, as characterized by fewer frequencies of critical and reflective use and appraisal of web-based health information in terms of their credibility, trustworthiness, and applicability in personal circumstances. Limited CRHL was also defined by less engagement in community-level health promotion and disease prevention activities, as well as less importance attached to the quality of health information and healthy lifestyles, in comparison with factors such as housing conditions, income, jobs, and local facilitates. The highest average sum scores of participants in the second latent class suggest that this subgroup of the study populations had the lowest CRHL, in comparison with class 1 (mean 9.19, SD 1.33) and class 3 (mean 11.72, SD 0.91), which we labeled as adequate and moderate CRHL groups, respectively. We then continued to explore the factors associated with low CRHL (class 2) among the Chinese study participants using logistic regression modeling (forward stepwise), and the principal findings are summarized in subsequent sections.

#### Factors Associated With the Participants’ Low CRHL

##### Older Age

LCA showed that the posterior probabilities of cluster 2 were the highest among the study participants aged between 41 and 50 and between 51 and 68 years, as shown in Table 7. In contrast, people aged between 35 and 40 years had the highest posterior probabilities of belonging to cluster 1 of adequate CRHL, and people aged between 17 and 34 years had the highest posterior probabilities of belonging to cluster 3 of moderate CRHL. This finding suggests that middle-aged and older adult Chinese were least likely to critically assess web-based health information, reflect on the credibility of web-based resources, and question health professionals based on their research, and they were less active in voicing their opinions about what the government does about health (CRHL5). They also gave stronger importance to external conditions such as income and health facilities than to quality health information and healthy lifestyles (CRHL6), as shown in Table 4.

**Table 7:** Posterior probabilities^a^ of age groups across the latent clusters.

Age (years)	Cluster 1	Cluster 2	Cluster 3	Total probability
17-28	0.37	0.23	0.41	1
29-34	0.33	0.33	0.34	1
35-40	0.44	0.24	0.32	1
41-50	0.36	0.43	0.20	1
51-68	0.39	0.43	0.17	1

^a^A posterior probability, in Bayesian statistics, is the revised or updated probability of an event occurring after considering new information. The posterior probability is calculated by updating the prior probability using the Bayes theorem. In statistical terms, the posterior probability is the probability of event A occurring, given that event B has occurred.

##### The Male Sex

The results of the analysis of posterior probabilities of sex across the latent clusters in our study showed that male participants were more likely to fall into the limited CRHL class (cluster 2) with a probability of 40%, rather than into the adequate CRHL class (cluster 1) with a probability of 38% and the moderate CRHL class (cluster 3) with a probability of 22%. In contrast, female participants were more likely to fall into the adequate CRHL class (cluster 1) with a probability of 38%, rather than into the limited CRHL class (cluster 2) with a probability of 30% and the moderate CRHL class (cluster 3) with a probability of 33%.

##### Limited Education (Year 6-Year 12)

We found that lower educational attainment (Year 6-Year 12) was a significant predictor of limited CRHL, as shown by cluster 2 in Table 8.

**Table 8:** Posterior probabilities of educational levels across the latent clusters.

Education	Cluster 1	Cluster 2	Cluster 3	Total probability
Year 6	0.28	0.55	0.16	1.00
Year 9	0.33	0.50	0.18	1.00
Year 12	0.32	0.54	0.14	1.00
Diploma	0.40	0.25	0.35	1.00
University	0.44	0.17	0.39	1.00
Postgraduate degree	0.43	0.10	0.47	1.00

##### Low Internal Drive to Manage One’s Health

When patients had very low scores on the Internal subscale of MHLC, that is, <17, which indicated low internal drives to manage one’s health, they were more likely to belong to cluster 2, with low CRHL, as shown in Table 9.

**Table 9:** Posterior probabilities of the Multidimensional Health Locus of Control (MHLC) internal subscale sum across the latent clusters.

MHLC—internal sum^a^	Cluster 1	Cluster 2	Cluster 3	Total probability
6-16	0.28	0.54	0.18	1
17-19	0.42	0.39	0.19	1
20-22	0.38	0.35	0.28	1
23-26	0.39	0.24	0.37	1
27-36	0.44	0.12	0.43	1

^a^Sum scores were computed by adding the scores of items 1, 6, 8, 12, 13, and 17 of the MHLC scale Form A.

## Discussion

### Principal Findings in Relation to Previous Studies

Using latent class modeling, we identified 3 latent classes among Chinese study participants, and the classes were labeled as limited, moderate, and adequate CRHL groups. Four factors were ascertained to be associated with low CRHL (class 2) among the Chinese study participants, as summarized in the principal findings in subsequent sections.

#### Principal Finding 1: Low CRHL Was Prevalent Among People Aged Between 41 and 68 Years

This finding confirms the findings in many relevant studies. As found by Manganello [42], health literacy may be predicted by age. Baker et al [43] reported a similar finding that FUHL was markedly lower among older age groups and that there was an association between increasing age and lower FUHL. This association was additionally cited by Rudd [44] and Paasche-Orlow et al [6], who revealed that older patients and patients who are less educated are more likely to have lower health literacy. The association between increasing age and lower health literacy may in part be explained by age-related decline in cognitive function [43,45]. As such, “addressing health literacy at an early age can help develop one’s ability to understand health information and improve interactions with the health care system that will lead to positive health outcomes later in life” [42].

However, Tschaftary et al [46] discovered that the older the patients were, the more health literate they were. This opposite finding warrants further studies to ascertain the positive or negative association between older age and lower health.

#### Principal Finding 2: Male Participants Were More Likely to Have Low CRHL

This finding parallels the finding reported by Kaneko and Motohashi [47] that poor mental health literacy was strongly associated with male sex and lower educational attainment. Similarly, as reported by Clouston et al [48] predictors of low health literacy included lower levels of educational attainment and male sex. These findings reinforce the findings of Lee et al [49], who found that Korean female individuals had higher health literacy than Korean male individuals, and the findings of Kim [50], who reported that health literacy levels were higher in male individuals. However, the study by Kim et al [51] revealed that health literacy was significantly higher in Korean male individuals. This inconsistency can also be found in studies carried out by Kunter et al [52] and Paasche-Orlow et al [6] among American populations. The mixed findings concerning the association between sex and health literacy can add important information to the growing understanding of the role of sex in health literacy [49]. However, these inconsistent findings warrant further studies that are to be conducted in diverse linguistic, cultural, ethnic, and socioeconomic communities to further scrutinize the correlation between sex and health literacy status.

#### Principal Finding 3: People With Limited Education (Year 6-Years 12) Were Likely to Have Low CRHL

Rudd [44] and Paasche-Orlow et al [6] reported similar findings that less-educated patients tended to have limited health literacy. These findings reinforce those of Kaneko and Motohashi [47] and Clouston et al [48], who reported that limited mental health literacy was strongly associated with lower educational attainment. Similarly, as found in other studies, the level of education is more consistently associated with the level of health literacy [6,53,54]. In contrast, it has also been found that higher health literacy was associated with higher educational attainment [49]. These previous studies, together with our study, may add to the growing body of evidence for the role of limited educational attainment in predicting poor health literacy.

#### Principal Finding 4: People With a Low Internal Drive to Manage Their Health Were Likely to Have Low CRHL

The predictive role of low internal drive in managing one’s health has not been investigated in the literature, to the best of our knowledge, based on our retrieval of relevant studies in the existing literature. Therefore, we could not compare this finding with those of previous studies. This gap in the literature needs to be addressed in future research.

### Implications

This study adds to the limited body of literature on CRHL and its associated factors. The findings can provide some implications for clinical practice, health education, medical research, and public health policy making. The 3 CRHL classes and 4 predictors of limited CRHL may serve as important indicators for screening Chinese people with limited CRHL to deliver more targeted education and effective interventions. Knowledge, skills, beliefs, and practices associated with the 4 ascertained predictors could be integrated into public health education and interventions in CRHL among the Chinese population. Medical researchers can gain certain insights into the topic of limited CRHL and its associated factors. Informed by this study, they could identify populations with limited CRHL among their ethnic and socioeconomic groups, verify the factors ascertained in this study, and identify more contributors in future research.

### Limitations

This study has some limitations. The first limitation concerns the generalizability of our research results and findings. The recruitment of patients from only one hospital may make the results and findings less generalizable to populations in other provinces in China and different linguistic and cultural communities worldwide. Further research is warranted to validate the results and findings among populations with diverse ethnic and sociocultural backgrounds. Second, self-reported responses from the participants may incur some bias. As found by Van der Varrt et al [55], self-reported literacy skills are not necessarily consistent with the actual ability to comprehend, use, and appraise web-based health information. This is true for the self-reported literacy skills on other scales and the self-reported health beliefs and self-confidence on the MHLC scales Form A used in this study. More objective measures need to be developed to increase the reliability and consistency of assessments of various health literacy, health beliefs, and self-confidence among culturally and linguistically diverse people.

### Conclusions

Using latent class modeling, we identified 3 classes of CRHL (ie, limited, moderate, and adequate) among Chinese study participants and 4 factors associated with limited CRHL: (1) middle and old age, (2) male sex, (3) lower educational attainment, and (4) low internal drive to maintain one’s health. These literacy classes and predicting factors ascertained in this study can provide some implications for clinical practice, health education, medical research, and health policy making.

## Appendix

Multimedia Appendix 1 Questionnaire and collected data.

## Abbreviations

AAHLS: All Aspects of Health Literacy Scale
AIC: Akaike information criterion
BIC: Bayesian information criterion
COHL: communicative health literacy
CRHL: critical health literacy
eHEALS: eHealth Literacy Scale
FUHL: functional health literacy
GHNT: General Health Numeracy Test
LCA: latent class analysis
MHLC: Multidimensional Health Locus of Control

## Data Availability

Data are available in Multimedia Appendix 1.
